# CD169 Expression in Lymph Nodes is Associated with Increased Infiltration of CD8^+^ T Cells in Tumors: A Systematic Review and Meta-Analysis

**DOI:** 10.1155/2024/8873767

**Published:** 2024-01-12

**Authors:** Yong Wang, Xiao-Ting Wu, Jing Chen

**Affiliations:** ^1^Department of Gastrointestinal Surgery, West China Hospital, Sichuan University, Chengdu, China; ^2^Healthcare-Associated Infection Control Center, Sichuan Provincial People's Hospital, University of Electronic Science and Technology of China, Chengdu, China

## Abstract

The density of CD169^+^ macrophages has been reported to positively correlate with the number of CD8^+^ T cells, although this remains controversial. To better understand this topic, we conducted a meta-analysis. We searched the PubMed, Medline, and Web of Science databases for studies that were published before May 2022 and performed a meta-analysis of the incidence of low and high CD169 expression in groups based on CD8 expression using the random-effects model. A total of 10 studies were included in the meta-analysis. The incidence of high CD169 expression in lymph nodes was significantly lower than that of low CD169 expression in the low CD8 expression group (odds ratio (OR): 0.76, 95% confidence interval (CI): 0.6, 0.96); however, the incidence of high CD169 expression in lymph nodes was higher than that of low CD169 expression in the high CD8 expression group (OR: 1.50, 95% CI: 1.08, 2.07). We also found that the expression of CD169 in tumors was lower than that in nontumor tissues (standardized mean difference: −5.29, 95% CI: −7.47, −3.11). The overall survival and hazard ratio of patients with high and low CD169 expression was 0.45 (95% CI: 0.37, 0.55). This analysis showed that high CD169 expression was associated with a high CD8 expression, and low CD169 expression was associated with low CD8 expression. The risk of death was 55% lower for patients with high CD169 expression, and high CD169 expression may be associated with favorable survival outcomes in cancer patients. However, the number and heterogeneity of the studies should be taken into consideration when evaluating the analysis. High-quality randomized controlled trials on the association between CD169 and CD8 expression are needed to verify these effects.

## 1. Introduction

Tumor progression can generate a more supportive microenvironment that facilitates escape from the host immune system in various ways. This immunosuppressive environment limits the effectiveness of anticancer chemotherapy. The generation of tumor antigen-specific cytotoxic T lymphocytes (CTLs) is considered the key to antitumor immunity. Tumor-infiltrating CD8^+^ lymphocytes are involved in anticancer immune responses, and a high density of CD8^+^ lymphocytes in tumors is associated with a favorable prognosis for some cancers [1–4]. Lymph nodes play an important role in inducing specific immune responses to cancer [5, 6]. Various antigens flow into lymph nodes, where dendritic cells and macrophages act as antigen-presenting cells [7, 8]. Lymph node sinus macrophages (LySMs) are also known to have antigen-presenting capacity in animal studies. CD169, also called sialoadhesin, is found in LySMs. The downregulation of CD169 in regional lymph nodes was associated with lymph node metastasis in a rat model, and CD169^+^ lymph node macrophages have protective functions against mouse breast cancer metastasis [9, 10]. CD169^+^ LySMs, as well as dendritic cells, were involved in antigen presentation and the induction of CTLs in a mouse model [11, 12]. It has been reported that CD169^+^ macrophages can enhance antitumor immunity in mice by cross-presenting tumor antigens to CD8^+^ T cells [11, 13]. Recently, some studies have examined the role of LySMs in patients with various cancers. We analyzed the association between CD169 expression and CD8 expression. Some studies found that the density of CD169^+^ macrophages positively correlated with the number of CTLs [14, 15]. However, another study reported no significant correlations between CD169^+^ macrophage cell density and the density of CD8^+^ lymphocytes [16]. There is no consensus on this issue, nor has there been a published systematic study. To better understand this topic, we conducted this meta-analysis.

## 2. Materials and Methods

### 2.1. Search Strategy

We performed a search of the PubMed, Medline, and Web of Science databases. The final search was conducted in May 2022, and the search terms included were as follows: (CD169 or CD169^+^ cell or CD169^+^ macrophages) and (CD8 or CD8^+^ T cell or cytotoxic T cell) and (cancer or tumor or malignancies). The reference list of each paper was scanned to identify additional studies. If necessary, we contacted the authors for more information.

### 2.2. Selection Criteria

Studies were included if they met the following criteria: clinical data from patients, data on CD169 interacting with CD8 T cells or data necessary to assess it, and the effect of CD169 in patients with cancer.

### 2.3. Exclusion Criteria

Studies were excluded according to the following criteria: no clinical data from patients with cancer, no data on CD169 interacting with CD8^+^ T cells or data necessary to assess it, or duplicated data.

### 2.4. Data Extraction

Three reviewers (YW, JC, and XTW) extracted all the data independently according to the selection criteria. The articles were discussed again in case of divergent opinions. The following information was extracted: patient age, sample size, tumor type, CD169 and CD8 expression location, detection methods, and classification of high- and low-density expression.

### 2.5. Statistical Analysis

We performed all statistical analyses with Statistical Software-STATA, version 12.0. The expression of CD169 in intratumoral and nontumor tissues was pooled using the fixed-effects model with a mean difference. We also analyzed the incidence of low and high CD169 expression in different CD8 expression groups using the random-effects model. The measure of the effect of interest is the odds ratio (OR) with a 95% confidence interval (CI). We used the *Q* and *I*^2^ statistics to test the statistical heterogeneity of the studies [17]. A *P* value of <0.1 was considered indicative of statistically significant heterogeneity for the *Q* statistic. Heterogeneous studies were excluded. Data synthesis of these heterogeneous studies was presented in a narrative analysis. The Egger weighted regression method was used to assess publication bias [18]; a *P* value of <0.1 indicated statistically significant publication bias.

## 3. Results

### 3.1. Search Result

**Figure 1:**
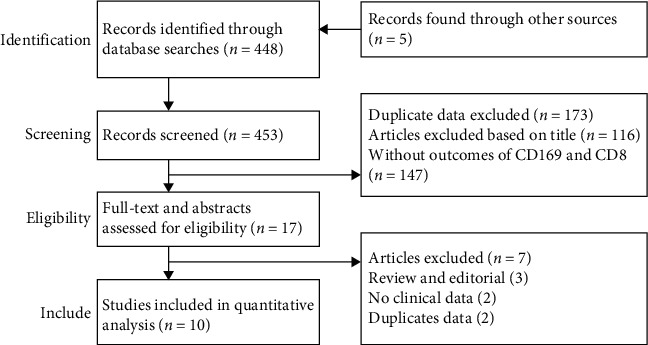
Screening and selection process for the studies.

We identified 453 articles in the search and screened their titles and abstracts. Only 17 articles were considered eligible. After a review of the full-text articles, 10/17 articles met the inclusion criteria and were eligible for this meta-analysis.  Figure 1 shows the selection process.

### 3.2. Baseline Characteristics

Ten studies [14–16, 19–25] were included, which were published between 2015 and 2021. The characteristics of these studies are shown in Table 1. The sample sizes of the studies ranged from 44 to 294 cases. These studies reported the association between CD169^+^ macrophages and CD8^+^ T cells in patients with gastric cancer, hepatocellular carcinoma, colorectal carcinoma, esophageal cancer, bladder cancer, endometrial carcinoma, malignant melanoma, breast cancer, or oral squamous cell carcinoma. Seven studies examined the expression of CD169 in lymph nodes, and two studies reported expression in intratumor and nontumor tissues. All of the studies examined the expression of CD169 and CD8 through immunohistochemistry. The numbers of patients in all of the studies according to different CD169 and CD8 expression levels are shown in Table 2.

### 3.3. Overall and Stratified Analysis

**Figure 2:**
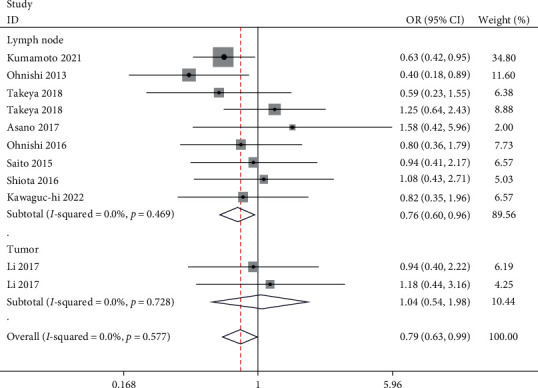
Forest plot for high CD169 expression and low CD169 expression in patients with low CD8 expression.

**Figure 3:**
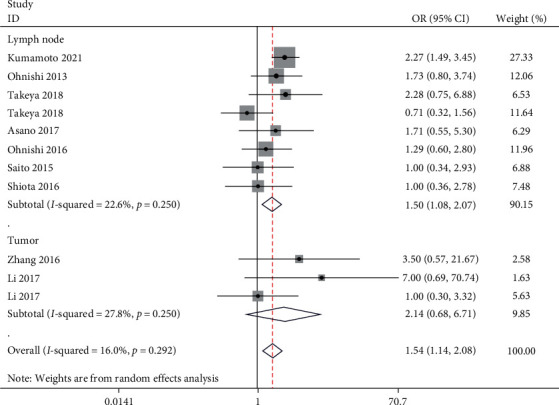
Forest plot for high CD169 expression and low CD169 expression in patients with high CD8 expression.

We performed a meta-analysis of the studies for the incidence of low and high CD169 expression based on different CD8 expression levels; two studies included data for CD169 expression in the tumor. We excluded the Zhang et al. [20] study through sensitivity analysis, which did not alter the outcome of the analysis. The meta-analysis showed that the incidence of high CD169 expression in lymph nodes was significantly lower than that of low CD169 expression in the group with low CD8 expression (OR: 0.76, 95% CI: 0.6, 0.96); however, there was no significant effect of high or low intratumoral CD169 expression (OR: 1.04, 95% CI: 0.54, 1.98) (Figure 2). We also excluded the Kawaguchi et al. [25] study through sensitivity analysis and found that the incidence of the high CD169 expression in lymph nodes was greater than that of low CD169 expression in the high CD8 expression group (OR: 1.50, 95% CI: 1.08, 2.07); there was no significant effect of different intratumoral CD169 expression levels (OR: 2.14, 95% CI: 0.68, 6.71) (Figure 3). This analysis also found that the expression of CD169 in tumors was lower than that in nontumor tissues (standardized mean difference: −5.29, 95% CI: −7.47, −3.11). There was slight heterogeneity in the analysis of CD169 expression in tumors (*I*^*2*^ = 22.6%) and in lymph nodes for the high CD8 group (*I*^*2*^ = 27.8%); however, the other analysis did not show significant heterogeneity.

### 3.4. Analysis of Overall Survival (OS) of Patients with High CD169 Expression

**Figure 4:**
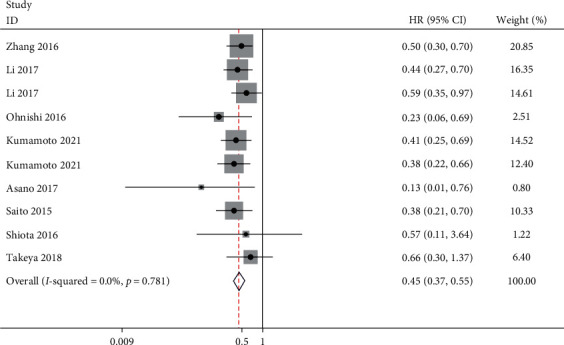
Forest plot for overall survival of patients with high CD169 expression.

This analysis excluded the Ohnishi et al. [14] and Kawaguchi et al. [25] studies through sensitivity analysis. The hazard ratios for OS of patients with high and low CD169 expression were 0.45 (95% CI: 0.37, 0.55) (Figure 4). Namely, the risk of death was 55% lower for the group with high CD169 expression. There was no heterogeneity revealed by the analysis.

### 3.5. Publication Bias

The Egger weighted regression method indicated that there was no publication bias in the analysis of the incidence of different CD169 expression levels in the low and high CD8 expression groups or for OS in the high CD169 expression group (*P*=0.151, 0.19,  and 0.237, respectively).

## 4. Discussion

CD169 expression is considered a surrogate marker of active immune responses in lymph nodes [16]. CD169^+^ macrophages are innate immune cells that limit the spread of pathogens by phagocytosis and degradation. CD169^+^ macrophages that reside in the lymph node sinus take up dead tumor cells and directly cross-present tumor antigens to CTLs. Mice lacking CD169^+^ macrophages at the time of dead tumor cell vaccination or chemotherapy-induced tumor degradation fail to induce antitumor immunity [11]. Macrophages are located at strategically important entry points, such as the subcapsular sinusoids of lymph nodes and the marginal and red medullary regions of the spleen, where they capture and filter pathogens [26]. CD169^+^ macrophages, which can be considered antigen-presenting cells, are important for CTL responses [12]. The expression of CD169 positively correlates with the density of CD8^+^ cytotoxic T cells. CD169^+^ macrophages significantly enhance T-cell proliferation, CD8^+^ cytotoxicity, and cytokine production in a CD169-dependent manner; further, autocrine TGF-*β* produced by tumor-stimulated macrophages is involved in downregulating CD169 expression [20]. In addition, CD169^+^ macrophages are involved in protumor antibody production [27] and are potentially associated with the efficacy of anti-PD-1/PD-L1 therapy, since they highly express PD-L1 [28]. There is a central role for CD169^+^ macrophages in the activation of acquired immunity. The participation of CD169^+^ cells in antigen presentation could be beneficial due to their localization at sites that are exposed to blood- and lymph-borne antigens that reach lymph nodes hours before migratory dendritic cells [13]. Recently, in a meta-analysis, Kong et al. [29] found that high expression of CD169 in the regional lymph node is associated with favorable survival outcomes in patients with malignant tumors and that CD169 may be a new, effective prognostic marker for malignancies.

We found that CD169 expression in tumors was lower than in nontumor tissues, based on a meta-analysis of two studies. The size of the meta-analysis should be taken into consideration when evaluating the findings. High-quality randomized controlled studies comparing CD169 expression in intratumoral sites and nontumor tissues are needed to verify this effect. However, we divided the patients into a group with high CD8 expression and a group with low CD8 expression, based on CD8 expression levels in the tumor, and performed a meta-analysis for different CD169 expression levels based on CD8 expression. We found that high CD169 expression in the lymph node was associated with a high density of CD8^+^ T cells and that low CD169 expression was associated with low CD8^+^ T-cell density, which is consistent with many studies in vitro and some animal experiments. CD169^+^ macrophages have been implicated in the activation of CD8^+^ T cells through two potential mechanisms: (i) antigen transfer to CD8*α*^+^ dendritic cells in the spleen [30] and (ii) direct antigen presentation to CD8^+^ T cells [11, 31]. During interaction with CD8^+^ T cells, CD169^+^ macrophages can themselves be targeted by activated CD8^+^ T cells. This has been reported in the case of splenic CD169^+^ macrophages after infection with *Plasmodium chabaudi* [32] and proposed in the case of subcapsular sinus macrophages after *Toxoplasma gondii* infection [31]. However, the expression of CD169 in tumors is not associated with CD8 expression. In our analysis, the studies used different standards to divide cases into high- or low-density groups for CD169 and CD8 expression. This may account for the significant heterogeneity in this study. Owing to the few studies in our analysis, significant heterogeneity was observed, especially for the analysis of the CD169 expression in the high-density CD8 group. RNA sequencing of CD169^+^ macrophages indicated that CD169^+^ macrophages were activated in hepatic TME and engaged in phagocytosis and immune modulation, probably as antigen-presenting cells, as reported [33], and hepatic CD169^+^ macrophages might raise accumulation of NK and T cells through secreting chemokines, such as CCL7 and CXCL14, and thus strengthening antitumor immune responses [34].

Some limitations of our study should be taken into consideration. First, we included some trials that had different evaluation criteria for CD169 and CD8 expression, which may influence the accuracy of the overall results. Second, the number of studies is relatively limited, which may cause problems in the evaluation of heterogeneity and publication bias, thereby reducing confidence in the results. Third, our study included studies with different kinds of cancers, which may influence the accuracy of the outcomes.

## 5. Conclusion

CD169 expression in the lymph node may be associated with a high density of CD8 expression in the tumor. In particular, high CD169 expression was associated with a high density of CD8 expression, whereas low CD169 expression was associated with low CD8 expression. The risk of death was 55% lower for patients with high CD169 expression, and high CD169 expression may be associated with favorable survival outcomes in cancer patients. However, the outcomes in this meta-analysis were based on a few studies, and some of the analyses had a high degree of heterogeneity. Thus, the size of the meta-analysis and the heterogeneity should be taken into consideration when evaluating the results. High-quality randomized controlled trials on CD169 and CD8 expression are needed to verify these effects.

## Figures and Tables

**Table 1:** Characteristics of included studies.

Study	Cancer	Follow-up	Number (year)	CD169 expression	CD169 detection	CD169 low/high	CD8 expression	CD8 detection	CD8 low/high
Kumamoto et al. [19]	GC (TNM (I–III/IV: 270/24)	144 months	294 (<70, ≥70)	LySMs in RLN LN-met: Neg: 175 Pos: 119	IHC	CD169^+^/CD68^+^ <0.65, ≥0.65	Tumor	IHC	CD8^+^/mm^2^ <287, ≥287

Zhang et al. [20]	HCC (TNM (I–II/III–IV: 227/144)	96 months	375 (20–81)	Tumor	IHC, FACS	CD169^+^/mm^2^ <200, ≥200	Tumor	IHC, FACS	CD8^+^/mm^2^ <150, ≥150

Ohnishi et al. [14]	CRC (TNM (I–II/III–IV: 49/34)	100 months	83 (29–90)	RLN LN-met: Neg: 55 Pos: 28	IHC	CD169^+^/mm^2^ <150, ≥150	Tumor	IHC	CD8^+^/mm^2^ <160, ≥160

Takeya et al. [21]	EPC (PT+, PT−) (TNM (I/II/III/IV 69/63/41/9)	140 months	182 (66.46)	LySMs in RLN LN-met: Neg: 95 Pos: 87 (cancer cell free)	IHC	CD169 score ≤4, >4	Tumor	IHC	CD8 score 1; 2 or 3

Li et al. [22]	HCC (TNM (I/II/III130/16/39) GC (TNM (I/II/III8/25/99)	120 months	188 (13–76) 138 (28–78)	Tumor	IHC	CD169^+^/mm^2^ <200, ≥200	Intratumor Nontumor	IHC	CD8^+^/mm^2^ <150, ≥150

Asano et al. [23]	BC (T1, T2: 22 T3, T4: 22)	140 months	44 (<70, ≥70)	RLN LN-met: Neg: 38 Pos: 6	IHC	CD169 score 0–4, 5–6	Tumor	IHC	CD8^+^/mm^2^ <343, ≥343

Ohnishi et al. [15]	EC (TNM (I/II–IV: 41/38)	120 months	79 (<60, ≥60)	RLN LN-met: Neg: 66 Pos: 13	IHC	CD169^+^/mm^2^ <350, ≥350	Tumor	IHC	CD8^+^/mm^2^ <120, ≥120

Saito et al. [16]	MM (TNM (I/II/III/IV 26/37/26/1)	100 months	95 (34–91)	RLN LN-met: Neg: 61 Pos: 23	IHC	CD169^+^/mm^2^ <300, ≥300	Tumor	IHC	CD8 score 1; 2 or 3

Shiota et al. [24]	Breast cancer (TNM (I/II–III: 57/89)	160 months	146 (<55, ≥56)	RLN LN-met: Neg: 92 Pos: 54	IHC	CD169^+^/mm^2^ <400, ≥400	Tumor	IHC	CD8^+^/mm^2^ <150, ≥150

Kawaguchi et al. [25]	OSCC (T1, T2 : 44 T3, T4 : 45)	70 months	89 (33–88)	RLN LN-met: Neg: 27 Pos: 62	IHC	CD169 score 0–4, 4.5–6	Tumor	IHC	CD8^+^/mm^2^ <1,000, ≥1,000

TNM: tumor-node-metastasis; LN-met: lymph node-metastasis; Neg: negative; Pos: positive; GC: gastric cancer; HCC: hepatocellular carcinoma; CRC: colorectal carcinoma; EPC: esophageal cancer; BC: bladder cancer; EC: endometrial carcinoma; MM: malignant melanoma; OSCC: oral squamous cell carcinoma; LySMs: lymph node sinus macrophages; RLN: regional lymph nodes; IHC: immunohistochemistry; FACS: flow cytometry; PT: pretreatment.

**Table 2:** The number of patients in different CD169 and CD8 expression.

Study		Low CD169 (*N*)	High CD169 (*N*)	CD169 (IT vs. NT)	HR for OS in high CD169 (95% CI)
Kumamoto et al. [19]	Low CD8	90/147	57/147	NR	Total GC: 0.41 (0.25–0.69)
	High CD8	45/147	102/147		Advanced GC: 0.38 (0.22–0.66)

Zhang et al. [20]	Low CD8	31/38	7/38	FACS (%) 45 ± 10.2 vs. 87.5 ± 5.6	0.50 (0.3–0.70)
	High CD8	2/9	7/9		

Ohnishi et al. [14]	Low CD8	30/42	12/42	NR	1.29 (0.72–2.39)
	High CD8	15/41	26/41		

Takeya et al. [21]	Low CD8	PT+: 22/29 PT-: 26/48	PT+: 9/20 PT-: 29/43	NR	0.662 (0.297–1.371)
	High CD8	PT+: 7/29 PT-: 22/48	PT+: 11/20 PT-: 14/43		

Li et al. [22]	Low CD8	HCC: 16/31 GC: 11/24	HCC: 15/31 GC: 13/24	IHC (%) HCC 30.4 ± 8.5 vs. 60.9 ± 14.3	0.436 (0.27–0.703)
	High CD8	HCC: 1/8 GC: 8/16	HCC: 7/8 GC: 8/16	GC 46.3 ± 8 vs. 88.4 ± 3.2	0.587 (0.354–0.974)

Asano et al. [23]	Low CD8	19/25	6/25	NR	0.13 (0.01–0.76)
	High CD8	7/19	12/19		

Ohnishi et al. [15]	Low CD8	20/36	16/36	NR	0.23 (0.06–0.69)
	High CD8	17/39	22/39		

Saito et al. [16]	Low CD8	17/33	16/33	NR	0.38 (0.21–0.70)
	High CD8	10/20	10/20		

Shiota et al. [24]	Low CD8	13/27	14/27	NR	0.57 (0.11–3.64)
	High CD8	11/22	11/22		

Kawaguchi et al. [25]	Low CD8	17/31	14/31	NR	3.009 (1.374–6.692)
	High CD8	11/58	47/58		

*N*: number; OS: overall survival; HR: hazard ratio; GC: gastric cancer; HCC: hepatocellular carcinoma; IT: intro-tumor; NT: nontumor; IHC: immunohistochemistry; FACS: flow cytometry; PT: pretreatment; NR: not reported.
